# Transient co-expression with three *O*-glycosylation enzymes allows production of GalNAc-*O*-glycosylated Granulocyte-Colony Stimulating Factor in *N. benthamiana*

**DOI:** 10.1186/s13007-018-0363-y

**Published:** 2018-11-06

**Authors:** Israel A. Ramírez-Alanis, Justin B. Renaud, Silverio García-Lara, Rima Menassa, Guy A. Cardineau

**Affiliations:** 1 School of Engineering and Sciences, Tecnologico de Monterrey, Campus Monterrey, Av. Eugenio Garza Sada 2501 Sur, C.P. 64849 Monterrey, NL Mexico; 2 Arizona State University, Phoenix, AZ 85004-4467 USA; 3 Agriculture and Agri-Food Canada, London, ON Canada; 4 Department of Biology, University of Western Ontario, London, ON Canada

**Keywords:** Mucin-type *O*-glycosylation, G-CSF, Granulocyte-Colony Stimulating Factor, *Nicotiana benthamiana*, Pharmaceutical glycoprotein, Molecular farming

## Abstract

**Background:**

Expression of economically relevant proteins in alternative expression platforms, especially plant expression platforms, has gained significant interest in recent years. A special interest in working with plants as bioreactors for the production of pharmaceutical proteins is related to low production costs, product safety and quality. Among the different properties that plants can also offer for the production of recombinant proteins, protein glycosylation is crucial since it may have an impact on pharmaceutical functionality and/or stability.

**Results:**

The pharmaceutical glycoprotein human Granulocyte-Colony Stimulating Factor was transiently expressed in *Nicotiana benthamiana* plants and subjected to mammalian-specific mucin-type *O*-glycosylation by co-expressing the pharmaceutical protein together with the glycosylation machinery responsible for such post-translational modification.

**Conclusions:**

The pharmaceutical glycoprotein human Granulocyte-Colony Stimulating Factor can be expressed in *N. benthamiana* plants via agroinfiltration with its native mammalian-specific mucin-type *O*-glycosylation.

**Electronic supplementary material:**

The online version of this article (10.1186/s13007-018-0363-y) contains supplementary material, which is available to authorized users.

## Background

Plants have emerged as alternative expression systems for the production of pharmaceutical proteins [1–4]. While there are several advantages to using plants as expression systems, such as low cost of production and maintenance, fast scalability, biological safety, and proper protein folding and assembly [1–3, 5], there are also some limitations concerning bioactivity or quality of the produced proteins [6]. Although plants are known to perform post-translational modifications, just like mammalian cells do, differences in glycosylation patterns between plant and animal cells might represent a major drawback that can impact the biochemical properties of the plant-derived recombinant protein [5–10].

With regard to the differences in glycosylation patterns between humans and plants, plant *N*-glycosylation differs mainly in the attachment of α(1,3)-fucose and β(1,2)-xylose to the core *N*-glycan, while *N*-glycans in human and mammalian cells present α(1,6)-fucose and no xylose is attached. Further maturation of the plant core *N*-glycan typically results in a biantennary structure, occasionally with terminal Lewis A epitopes. Terminal Lewis A epitopes are rarely observed in human proteins, but are widely distributed in plant glycoproteins. Maturation of human *N*-glycans will present a multi-antennary structure with two or more terminal branches, further extended with galactose and sialic (neuraminic) acid [4, 11]. Major efforts to humanize *N*-glycosylation patterns in plants have focused on retaining the recombinant protein in the ER to avoid further modification of the core *N*-glycan with α(1,3)-fucose, β(1,2)-xylose and Lewis A, abolishing expression of glycosyltransferases responsible for the attachment of α(1,3)-fucose and β(1,2)-xylose, and further humanization has included the expression of human β(1,4)-galactosyltransferase and heterologous enzymes required to generate sialylation, which are present in human cells but absent in plants [4, 11–14].

In the case of *O*-glycosylation, patterns differ significantly between plants and human cells, and specifically from the human mucin-type *O*-glycosylation. In plants, protein *O*-glycosylation is characterized by the presence of *O*-glycans attached to hydroxyprolines (Hyp) and Ser residues. Typically, Hyp residues in plants are decorated with arabinogalactan polysaccharides or arabinose oligosaccharides, and Ser residues with galactose [6, 11, 15, 16]. While human mucin-type *O*-glycosylation is characterized by the attachment of *N*-acetylgalactosamine (GalNAc) to Ser or Thr residues, which can be further elongated with other sugars to form specific and highly complex glycans. Although mucin-type *O*-glycosylation is widely present in human proteins, plants lack the enzymatic machinery to perform this specific glycosylation [11, 15, 16].

Recently, a few, but significant, advances in engineering *O*-glycosylation in plants to produce mucin-type glycans were reported. Among the recent achievements, Daskalova et al. [6] successfully expressed an *O*-glycosylated human Mucin 1 peptide derivative, which was detected exclusively as a glycoform in *Nicotiana benthamiana*. This was achieved by co-expressing the *Yersinia enterocolitica* UDP-GlcNAc 4-epimerase, in charge of converting UDP-GlcNAc to UDP-GalNAc in the cytoplasm, and a *Caenorhabditis elegans* UDP-GlcNAc/UDP-GalNAc transporter, responsible for the transport of the sugar donor to the Golgi lumen, together with the human GalNAc transferase 2.

In 2012, Yang et al. [10] were also able to generate mucin-type *O*-glycosylation in *N. benthamiana*, using a similar approach. They transiently expressed a *Pseudomonas aeruginosa* UDP-GlcNAc 4-epimerase, together with the human GalNAc-T 2 and 4, and a human 3.5 tandem repeat of Mucin1. The derivative mucin peptide was GalNAc-*O*-glycosylated with up to three and five GalNAc residues, when expressed with GalNAc-T2 and GalNAc-T 2 together with GalNAc-T 4, respectively. The mucin-type *O*-glycosylation was also demonstrated by mass spectrometry on a tandem repeat of MUC16 and on interferon α2b. These authors [17] were also able to establish mucin-type *O*-glycosylation in *Arabidopsis thaliana* and *Nicotiana tabacum* BY-2 cells, and encountered a high degree of proline hydroxylation and Hydroxyproline linked arabinosides, plant-specific *O*-glycosylation, on the substrate model protein.

The human Granulocyte-Colony Stimulating Factor (G-CSF) is a cytokine that stimulates the production, proliferation, differentiation and activation of neutrophil stem cells, raising the levels of neutrophils in the blood stream, and thus protecting the organism against bacterial, fungal and viral infections [18–22]. This cytokine is an important glycoprotein that is used as treatment in patients with neutropenia to reduce opportunistic infections, especially in cancer patients undergoing chemotherapy or radiotherapy [20, 23, 24]. Due to this clinical relevance, this protein is one of the most widely sold pharmaceuticals, thus making its production for clinical use very important [24–27]. G-CSF is mucin-type *O*-glycosylated at a single Thr residue. It has been successfully expressed in CHO cells, *Escherichia coli*, yeast and plants [28–32]. Nevertheless, only the mammalian platform variant is reported to be mucin-type *O*-glycosylated, while the yeast-derived variant is decorated with mannose residues. Several studies have shown the impact of glycosylation on protein stability [33–37], pointing out the relevance of production and correct glycosylation of this pharmaceutical.

In this project, our objective is to transiently express the human Granulocyte-Colony Stimulating Factor as a model pharmaceutical protein in *N. benthamiana* plants, via *Agrobacterium* infiltration, together with the genes required for the synthesis of *N*-acetylgalactosamine (GalNAc)-*O*-glycosylation, allowing the production of a plant-derived human-specific *O*-glycosylated G-CSF.

## Experimental procedures

### Construction of binary vectors

The coding sequence corresponding to the human G-CSF variant 2 (NCBI: NM_172219) was chemically synthesized (GenScript, Piscataway, NJ, USA) in three different fragments to build the variants used in this study. The three fragments consisted of the nucleotide sequences corresponding to the secretory signal peptide (pUC57 Nat-SP), the mature G-CSF fragment with a mutated glycosylation site that encodes an Ala, instead of the native Thr, and an extra 5′-end Met (pUC57 G-CSF A), and a 5′-end enterokinase coding sequence fused to the mature G-CSF fragment with the native glycosylation site (pUC57 EkG-CSF). The Sec-G-CSF coding sequence was built by digesting the pUC57 Nat-SP with NcoI and Bsu361 and using the digested fragment to replace the 5′-end EkG-CSF fragment of the pUC57 EkG-CSF, which was released with the same restriction enzymes, producing the pUC57 Sec-G-CSF. The Cyt-G-CSF coding sequence was built by releasing the 3′-end of the pUC57 G-CSF A with SapI cutting upstream the non-glycosylation site and downstream the stop codon. The same restriction enzyme was used to digest the pUC57 EkG-CSF, releasing the same fragment but containing the native glycosylation site (Thr), and the released fragment was cloned into the SapI opened pUC57 G-CSF A, generating the pUC57 Cyt-G-CSF with the glycosylation site. The Zera-G-CSF coding sequence was built by digesting the pUC57 EkG-CSF with NcoI and SacI, releasing the whole fragment, and ligating it into a pUC18 ZeraF1V vector available in the lab [38], by releasing the F1V fragment with the same enzymes and thus generating the pUC18 Zera-G-CSF. The different G-CSF coding sequences generated were then PCR amplified to introduce the flanking BbsI A (5′-end Sec-G-CSF: Mt114F—GGGGGGGAAGACATCATGGCTGGACCTGCCACTC), (5′-end Cyt-G-CSF: Mt23F—GGGGGGGAAGACATCATGACACCCTTAGGACC), (5′-end Zera-G-CSF: Mt20F—GGGGGGGAAGACGTCATGAGGGTGTTGCTCGTTGC) and B (3′-end of all G-CSF variants: M113R—CCCCCCGAAGACAGAGCTCGGGTTGAGCAAGGTGAC) sites, and removal of the stop codon to allow in frame fusion with the appropriate tag. The BbsI A site contains the recognition sequence for the restriction enzyme, followed by the specific overhang CATG, and the BbsI B site contains the specific overhang AGCT, allowing future GoldenGate cloning and disruption of the stop codon for further in-frame cloning with the respective tag. The resulting amplified product was cloned in pUC57 vector via EcoRV, and the mutated sites were verified by DNA sequencing and restriction digestions. The resulting pUC57 G-CSF variant without Stop codon (pUC57 Sec-G-CSF BbsI AB w/o Stop, pUC57 Cyt-G-CSF BbsI AB w/o Stop, pUC57 Zera-G-CSF BbsI AB w/o Stop) was then used to clone the coding sequence into the pENTR4 BbsI AB vector (a GoldenGate compatible pENTR4 vector generated in the lab), via GoldenGate, using the restriction enzyme BbsI and DNA ligase in a single cutting-ligation reaction. The resulting pENTR4 G-CSF variant without Stop codon (pENTR4 Sec-G-CSF w/o Stop, pENTR4 Cyt-G-CSF w/o Stop, pENTR4 Zera-G-CSF w/o Stop) was then used to clone the corresponding coding sequence into the binary vector via Gateway LR reaction, allowing the in-frame cloning of the G-CSF variant with the desired C-terminal tag, eYFP (pGWB 641) or c-Myc (pGWB 617) [39]. The binary vectors were used to transform *Agrobacterium tumefaciens* strain AGL-1.

Other constructs used in this work were the suppressor of post-transcriptional gene silencing p19 from *Cymbidium ringspot tombusvirus* [40] to increase accumulation levels; SecGFP (as protein secretion control), SecGFP:KDEL (for ER localization signal) previously published [41], and STtmd:mRFP (as Golgi marker for co-localization) [42]. *Agrobacterium* strain AGL-1 was used for pGWB 642, and *Agrobacterium* strain EHA105 was used for p19, GFP, and STtmd:mRFP constructs. The binary vectors used to generate *O*-glycosylation were as follows: pH7WG2 GNE, containing the *Y. enterocolitica gne* gene coding for UDPGlcNAc-4 epimerase; pH7WG2 GT, containing the *C. elegans* UDPGlcNAc/UDPGalNAc Transporter gene; and pH7WG2 GNT2, containing the human GalNAc Transferase 2 coding sequence, previously published [6]. All were electroporated into the *Agrobacterium* strain AGL-1.

### Agrobacterium infiltration

*Agrobacterium tumefaciens* strains were cultured to an optical density at 600 nm (OD_600_) of 0.5–0.8. The cells were then collected by centrifugation at 1000 g for 30 min. The pellets were resuspended in Agro-infiltration solution (3.2 g/L Gamborg´s B5 medium and vitamins, 20 g/L sucrose, 10 mM MES pH 5.6, 200 μM 4′-Hydroxy-3′-5′-dimethoxyacetophenone) to a final OD_600_ of 1.0, followed by incubation at room temperature for 1 h, with gentle agitation. The suspension was then used for needle-less infiltration of the abaxial leaf epidermis through the stomata of *N. benthamiana* plants [43].

### Confocal analysis

Protein subcellular visualization was determined by imaging the abaxial epidermal cells of leaf samples, with an Olympus LSM FV1200. Different lasers allowed for imaging of the different fluorescent tag fusion proteins. For GFP imaging, the tag was excited with a 488 Argon laser and detected at 500–545 nm. For eYFP imaging, the tag was excited at 515 and detected at 530–545 nm. For mRFP imaging, the tag was excited at 559 nm with a He/Ne laser and detected at 570–545 nm. The Imaris software (version 7.6.1, Bitplane Scientific Software, Bitplane, Zurich, Switzerland) was used to generate 3D images from z-stack confocal images. Line-sequential scanning mode was used for co-localization imaging.

### Preparation of total protein extracts

Four leaf discs (approximate fresh weight of 30 mg) from at least three biological replicates per sample were collected with a 7 mm diameter cork borer, put into a 2 ml tube with three 2.3 mm zirconia/silica beads (Bio Spec Products Inc, Cat. No. 11079125z) and frozen in liquid N_2_. Collected leaf discs were pulverized in a Mixer Mill (Retsch, Haan, Germany) for 1 min at 30 Hz, in previously frozen homogenizer blocks. Pulverized tissue was spun down for 1 min at 1000 g and 300 μL Protein Extraction Buffer (PBST0.1%, 2% PVPP, 1 mM EDTA pH 8.0, 1 mM PMSF, 1 μg/mL Leupeptin, 100 mM Sodium L-ascorbate) or Reducing Extraction Buffer (50 mM Tris, pH 8.0, 1% SDS, 20 mM DTT) were added to the sample. Samples were vigorously vortexed 3 times for 5 s and centrifuged at 20,000*g* for 15 min at 4 °C. Cleared supernatant was transferred to a new tube and TSP (Total Soluble Protein) was quantified using the Bio-Rad Bradford [44] protein assay reagent (Bio-Rad, Cat. No. 5000006).

### SDS-PAGE western blotting and lectin blot

Serological assays were performed by resolving the protein sample in a sodium dodecylsulphate-polyacrylamide gel electrophoresis (SDS-PAGE), and transferred to a PVDF or Nitrocellulose membrane. Recombinant proteins were detected with a 1:5000 dilution of the primary mouse anti-c-Myc monoclonal antibody (GenScript, Cat. No. A00864) or 1:5000 dilution of the primary rabbit anti-GCSF polyclonal antibody (GeneTex, Cat. No. GTX31157), and 1:3000 dilution of the goat anti-mouse IgG HRP-conjugated secondary antibody (Bio-Rad, Cat. No. 170-6516) or 1:3000 dilution of the goat anti-rabbit IgG-conjugated secondary antibody (Bio-Rad, Cat. No. 1706515). In the case of lectin blots, a 1:1000 VVA-HRP conjugate (EY Laboratories, Cat. No. H-4601-1) was used. Blotted membranes were visualized with the enhanced chemiluminescence (ECL) detection kit (GE Healthcare, Cat. No. RPN2232), following the manufacturer recommendations, and imaged with the DNR Bio-Imaging System MicroChemi (RANCOM A/S, Birkerød, Denmark). Band analysis was performed using the TotalLab TL 100 software (Nonlinear Dynamics, Durham, NC).

### Protein purification

Protein extract was transferred to a spin column, and anti-c-Myc beads were added to the column, following the manufacturer instructions (MBL International, Cat. No. 3305). The sample was incubated at 4 °C for 1 h, with gentle agitation end-over-end. The column was briefly centrifuged for 10 s, and the flow through was recovered. Three washes were performed by adding 200 μL of the washing buffer provided with the kit, and briefly centrifuging. Finally, 20 μL of elution peptide was added to the column, the sample was incubated for 5 min at 4 °C, and the purified protein was recovered by centrifuging the column for 10 s. Elution was repeated twice.

### Analysis of *O*-glycans by mass spectometry

SDS-PAGE excised bands were Trypsin/Chymotrypsin (6.25 mg/L each) digested [45] at 30 °C overnight. The peptide digests were analyzed using an Easy-nLC 1000 nano-flow system with a 100 µm × 2 cm Acclaim C18 PepMap™ trap column and a 75 µm × 15 cm Acclaim C18 PepMap™ analytical (Thermo Scientific, MA, USA) coupled to a Q-Exactive™ Quadrupole Orbitrap mass spectrometer (Thermo Scientific, MA, USA). The flow rate was 300 nL min^−1^ and 10 µL of the protein digest was injected. 97% mobile phase A (LC/MS Optima water, 0.1% formic acid) was decreased to 90% over 3 min. Peptides were eluted with a linear gradient from 10 to 35% mobile phase B (LC/MS Optima acetonitrile 0.1% formic acid) over 21 min followed by 35–90% over 3 min and maintained for 8 min. The nanospray voltage was set at 2.1 kV, capillary temperature 275 °C, and S-lens RF level 55. The Q-Exactive was operated in top 10 data-dependent acquisition mode with a full scan mass range of 400–2000 *m/z* at 70,000 resolution, automatic gain control (AGC) of 1e6 and maximum injection time (IT) of 250 ms. The MS/MS scans were acquired at 17,500 resolution, AGC of 2e5, maximum IT of 50 ms, intensity threshold of 8e4, normalized collision energy of 27 and isolation window of 1.2 *m/z.* Unassigned, singly and > 4 charged peptides were not selected for MS/MS and a 20 s dynamic exclusion was used. The Thermo .raw files were converted to mascot generic format using Proteowizard v2 [46] and the MS/MS scans were searched against the target/reverse human G-CSF amino acid sequence and the *N. benthamiana* proteome (Sol Genomics Network, accessed Jan 10th, 2015) using X! tandem [47] search algorithm operated from the SearchGUI v.2.35 [48] interface and processed in PeptideShaker v1.3.6 [49]. A 3 ppm precursor ion mass error and a 0.02 Da product ion error were used along with carbamidomethylation as a constant modification and oxidation of methionine, Hex(1)NAc(1) of Thr, HexNAc of Thr as variable modifications. A 1% FDR rate was used at the protein, peptide and peptide spectrum match level.

### Statistical analysis

The statistical analyses were performed with the Minitab 18 software (Minitab Inc., PA, USA). A one-way analysis of variance (one-way ANOVA) was performed followed by Tukey test to find significance differences between means (statistical difference was defined as *p* ≤ 0.05).

## Results

1. Transient expression of G-CSF in *N. benthamiana* leaves.

**Fig. 1:**
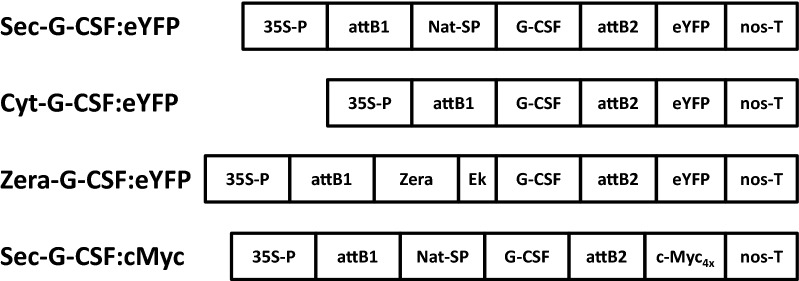
Schematic representation of constructs generated in this work and used for *Agrobacterium*-mediated transient expression in *N. benthamiana* leaves. Sec-G-CSF:eYFP, secretory G-CSF variant fused to C-terminal eYFP; Cyt-G-CSF:eYFP, cytoplasmic G-CSF variant fused to C-terminal eYFP; Zera-G-CSF:eYFP, G-CSF fused to N-terminal Zera peptide and C-terminal eYFP; Sec-G-CSF:cMyc, secretory G-CSF fused to C-terminal c-Myc_4x_. 35S-P, Cauliflower Mosaic Virus 35S promoter; nos-T, nopaline synthase transcription terminator; G-CSF, human Granulocyte-Colony Stimulating Factor coding sequence; Nat-SP, native secretory signal peptide from human G-CSF; Zera, γ-Zein ER-accumulating domain; Ek, enterokinase cleavage site; eYFP, enhanced yellow fluorescent protein for confocal and serological detection; c-Myc_4x_, four repeats of the c-Myc tag for detection and purification; *att*B1 and *att*B2, linker regions derived from recombination sites generated after Gateway cloning. Schematic representation not drawn to scale

To explore the feasibility of using *N. benthamiana* transient expression for the production of a mammalian glycoprotein, like G-CSF, several factors need to be explored, such as the accumulation levels of the recombinant protein, proper protein folding and native post-translational modifications. To address these issues, the native secretory human G-CSF (Sec-G-CSF) coding sequence, including its native secretory signal sequence, was used to build the corresponding expression cassettes, in order to target the model protein to the secretory pathway (Fig. 1). Two other G-CSF variants were also built, which would target the recombinant protein to the cytosol and as ER-derived protein bodies. These variants were used as controls to determine protein subcellular localization, as well as to evaluate the impact of protein accumulation. The cytoplasmic variant (Cyt-G-CSF) corresponds to the mature amino acid sequence with an N-terminal methionine, allowing the accumulation of the protein in the cytosol. The protein body variant (Zera-G-CSF) was achieved by fusing the N-terminus of the mature G-CSF coding sequence (no signal peptide) to the γ-Zein ER-accumulating domain: Zera^®^ peptide [38]. The different constructs were fused to an eYFP tag in order to track expression and proper subcellular localization of the recombinant protein. All of the G-CSF variant constructs-containing *Agrobacterium* strains were co-infiltrated with an *Agrobacterium* strain containing the p19 construct, a post-transcriptional gene silencing suppressor from *Cymbidium ringspot tombusvirus* (CymRSV) [40].

**Fig. 2:**
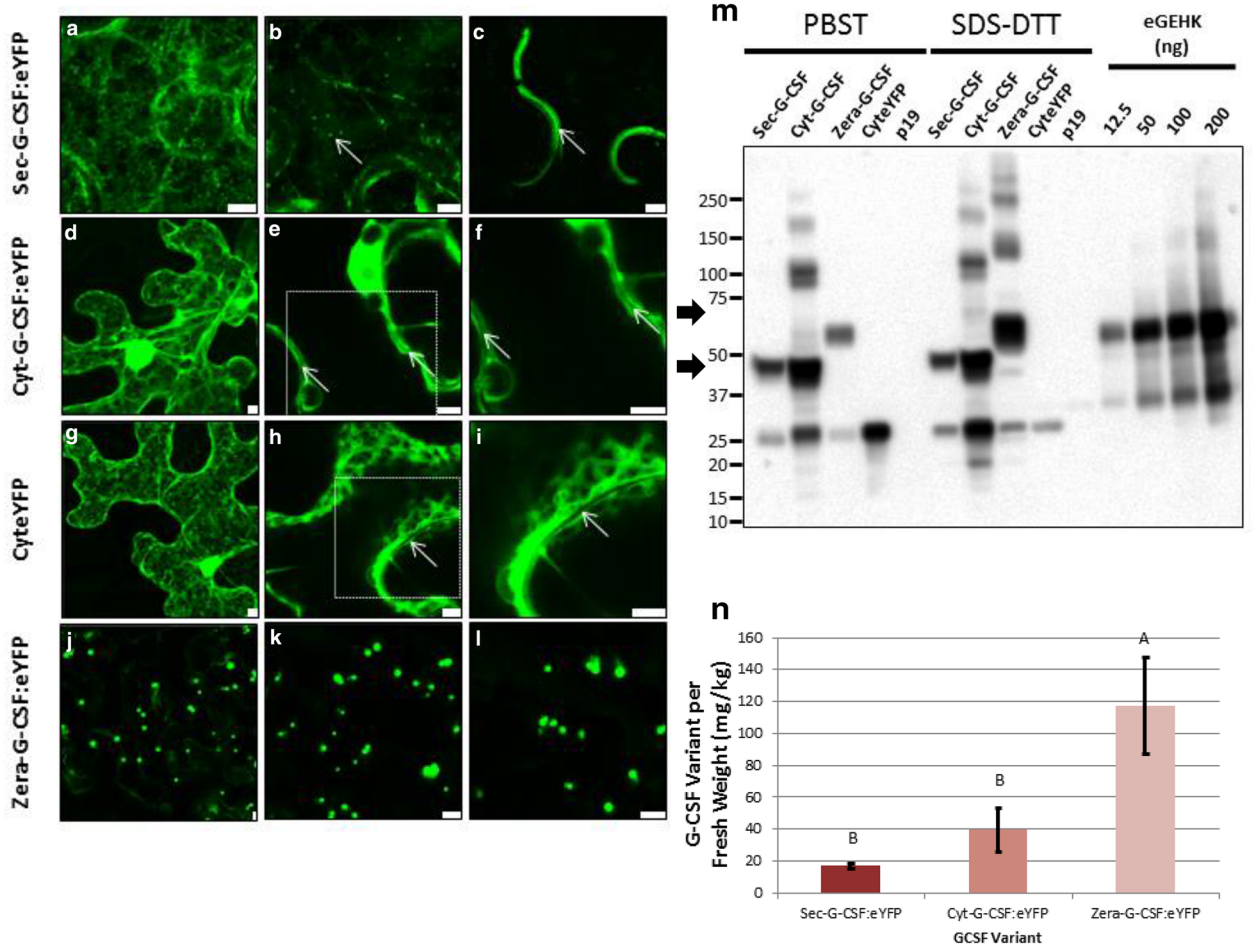
Transient expression of G-CSF:eYFP variants in *N. benthamiana* plants. Expression of Sec-G-CSF:eYFP (**a–c**), Cyt-G-CSF:eYFP (**d–f**), CyteYFP (**g–i**), Zera-G-CSF:eYFP (**j–l**). White arrows indicate apoplast localization (**c**) or absence of signal in the apoplast (**e, f, h, i**). White arrow indicating Golgi localization (**b**). Inset white boxes (**e**, **f**) depict microscopic zoom in shown in **f** and **i**, respectively. Samples collected at 4 dpi. Size bar: 5 μm. **m** Western Blot detection of Sec-G-CSF:eYFP (Secretory variant, 49 kDa), Cyt-G-CSF:eYFP (Cytoplasmic and mature protein variant, 49 kDa), Zera-G-CSF:eYFP (Zera fused variant, 61 kDa), eYFP (Cytoplasmic eYFP, 29 kDa). All samples were treated with PBS + Tween (PBST) extraction buffer and reducing extraction buffer (SDS-DTT). **n** Band quantification of SDS-DTT treated samples. 20 µg TSP of PBS samples were loaded on the gel. 9 µL (equalized volume to the smallest PBS sample amount) of SDS-DTT samples was loaded on the gel. Black arrows denote monomeric G-CSF:eYFP variants. eGEHK: protein standard. Proteins were detected with GFP antibody. Samples collected at 6 dpi. Columns denoted with a different letter are significantly different (*p* ≤ 0.05) using one-way ANOVA and followed by Tukey test. Error bars are standard deviation of the means

Expression of G-CSF:eYFP was first detected by confocal microscopy (Fig. 2a–l). Infiltrated *N. benthamiana* leaves were monitored from 2 to 8 dpi (days post-infiltration). Sec-G-CSF:eYFP was detected in the ER (endoplasmic reticulum), as a multiple dot-like pattern indicating a potential localization in the Golgi, as well as in the apoplast (Fig. 2a–c), suggesting localization in the secretory pathway. The cytoplasmic variant, Cyt-G-CSF:eYFP, was detected in the cytosol, and no signal was detected in the apoplast (Fig. 2d–f). Cytoplasmic eYFP (CyteYFP) was used as control for cytoplasmic accumulation pattern, also allowing the visualization of an apoplast with no presence of recombinant protein (Fig. 2g–i). Finally, the Zera-G-CSF:eYFP was found forming protein bodies due to the N-terminal fusion of the Zera^®^ peptide (Fig. 2j–l).

Identity of the transiently expressed G-CSF:eYFP recombinant proteins was further corroborated by western blot (Fig. 2m, n). Sec-G-CSF:eYFP was detected as a monomeric form with an apparent molecular weight of 49 kDa, which corresponds to the mature G-CSF:eYFP protein (after signal peptide cleavage). The same molecular weight was observed with Cyt-G-CSF:eYFP, confirming signal peptide processing of the Sec-G-CSF:eYFP. Furthermore, multimers were observed with Cyt-G-CSF:eYFP. In the case of Zera-G-CSF:eYFP a weak band was detected when protein extraction was performed with PBST (PBS + Tween), but the Zera^®^ fusion variant was properly extracted when the plant tissue sample was treated in denaturing and reducing conditions, using an SDS-DTT containing extraction buffer. Multimers were also observed with Zera-G-CSF:eYFP. eYFP cleavage was observed with all the different variants, and the cleaved eYFP band was corroborated using the CyteYFP expressing samples. It has to be noted that both Sec-G-CSF and Cyt-G-CSF were successfully extracted with PBST extraction buffer (Additional file 1: Fig. S3), but Zera-G-CSF could only be properly extracted with a reducing extraction buffer (Fig. 2; Additional file 2: Fig. S2).

**Fig. 3:**
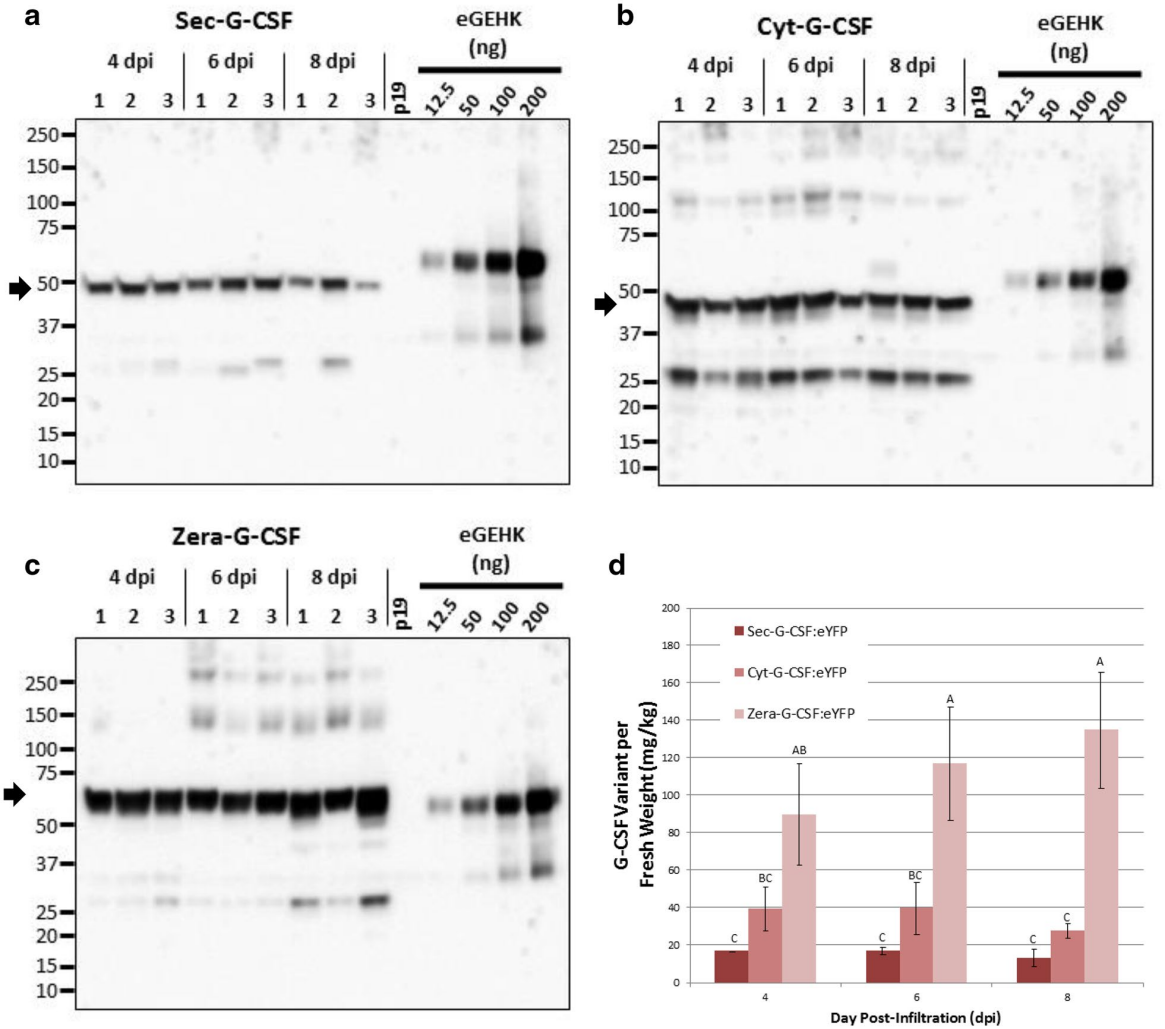
Time-course accumulation of transiently expressed G-CSF:eYFP variants in *N. benthamiana* plants. Western blot detection of Sec-G-CSF:eYFP (Secretory variant, 49 kDa) (**a**), Cyt-G-CSF:eYFP (Cytoplasmic and mature protein variant, 49 kDa) (**b**), Zera-G-CSF:eYFP (Zera fused variant, 61 kDa) (**c**). **d** Band quantification of Western blot detected proteins. Samples collected at 4, 6 and 8 dpi. Four leaf discs from three different plants were collected for each sample. All samples were treated with reducing extraction buffer (SDS-DTT). Equal amounts were used for all samples. Black arrows denote monomeric G-CSF:eYFP variants. eGEHK: protein standard. Proteins were detected with GFP antibody. Columns denoted with a different letter are significantly different (*p* ≤ 0.05) using one-way ANOVA and followed by Tukey test. Error bars are standard deviation of the means

G-CSF:eYFP accumulation was then assessed by densitometry analysis (Figs. 2n, 3a–d), with samples collected at 4, 6 and 8 dpi for each variant. We found that Sec-G-CSF reached an accumulation level of 17 mg/Kg F.W. (Fresh Weight), followed by Cyt-G-CSF:eYFP with 40 mg/Kg F.W., while Zera-G-CSF:eYFP reached 117 mg/Kg F.W., at 6 dpi (Figs. 2n, 3; Additional file 1: Fig. S3). We also observed that accumulation was stable at 4 and 6 dpi for Sec-G-CSF and Cyt-G-CSF, beginning to drop at 8 dpi, but not significantly (Fig. 3). There is no significant impact concerning efficiency of the extraction buffer of Sec-G-CSF (PBST or SDS-DTT) (Additional file 1: Fig. S3), but higher levels of accumulation were observed with Cyt-G-CSF when samples were treated with PBST, in comparison to SDS-DTT (Additional file 1: Fig. S3). Zera-G-CSF was the only variant that showed a significant increase in accumulation levels on the time-course, in comparison to the other two variants, reaching up to 135 mg/Kg F.W. at 8 dpi (Fig. 3), but as previously mentioned Zera-G-CSF could only be properly extracted under denaturing and reducing conditions using an extraction buffer containing SDS-DTT (Figs. 2, 3; Additional file 2: Fig. S2). This result was expected since previous studies indicate the usage of denaturing and reducing conditions for extraction of recombinant proteins fused to Zera [38, 50, 51]. The previous observations highlight the vulnerability of G-CSF to proteolytic degradation, and especially in the case of the secretory G-CSF.

2. Sec-G-CSF localization in the secretory pathway.

**Fig. 4:**
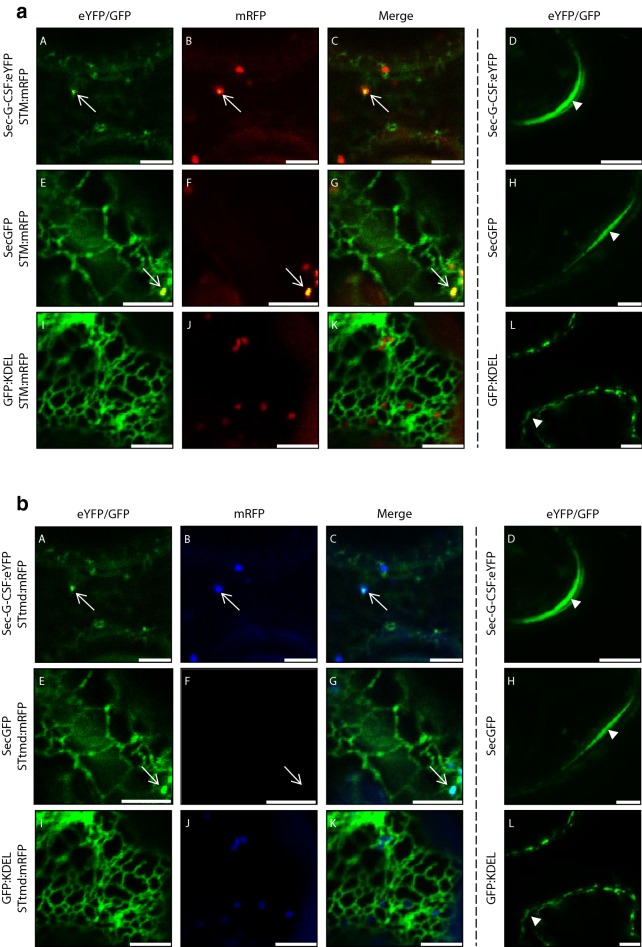
**a**, **b** Golgi and apoplast localization of Sec-G-CSF in *N. benthamiana* plants. Sec-G-CSF:eYFP (A, C, D), SecGFP (E, G, H), GFP:KDEL (I, K, L). Sec-G-CSF, SecGFP and GFP:KDEL were co-infiltrated with STM:mRFP (B, C, F, G, J, K). White arrows depict Golgi localization (A, B, E, F) and colocalization (C, G) with STM:mRFP. White arrowheads (images on the right) depict localization in apoplast (D, H), or apoplast without presence of recombinant protein (I). *Agrobacterium* AGL-1 was carrying Sec-G-CSF. SecGFP, GFP:KDEL, STM:mRFP and p19 were delivered by EHA105. 4 dpi. Size bar: 5 μm. **a** shows the colors as originally displayed, while **b** shows a color palette adjusted for those who have difficulty viewing red and green colors

To determine the feasibility of producing a mucin-type *O*-glycosylated G-CSF recombinant protein, we then corroborated that the native secretory signal peptide used in this work was targeting G-CSF to the secretory pathway. For this purpose, we determined the co-localization of Sec-G-CSF with Rat sialyltransferase, a Golgi resident enzyme, fused with monomeric red fluorescent protein (STtmd:mRFP) as a Golgi marker, which would suggest trafficking of secretory G-CSF along the secretory network (Fig. 4). Sec-G-CSF:eYFP was observed to localize in the ER as previously observed (Fig. 2) but also localized in the Golgi together with STtmd:mRFP (Fig. 4a–c). Although only a few Golgi bodies were captured where Sec-G-CSF would co-localize with the Golgi marker, a previously tested secretory GFP (SecGFP) construct from the lab [52] was used as a positive control for co-localization in the Golgi, and a similar pattern was observed, being SecGFP also localized in the ER (Fig. 4e–g). Thus, both Sec-G-CSF:eYFP and SecGFP were detected throughout the secretory pathway, in the ER, Golgi and in the apoplast (Fig. 4a–h). As a negative control for secretion, an ER-retained GFP (GFP:KDEL) construct was used, which would be directed to the secretory pathway but retrieved to the ER. The GFP:KDEL negative control was not found to co-localize with STtmd:mRFP, and it was not observed in the apoplast (Fig. 4i–k). The previous observations indicate that the native G-CSF secretory signal peptide indeed targets G-CSF to the secretory pathway. The same constructs were used to monitor apoplast signal, and only Sec-G-CSF:eYFP and SecGFP were observed in the apoplast, while GFP:KDEL was not, corroborating the previous observations (Fig. 4d, h, l). Although Sec-G-CSF was not exclusively observed in the Golgi, where *O*-glycosylation is traditionally referred to begin (Additional file 3: Fig. S1), the fact that the recombinant protein was observed in the apoplast suggested secretion of the protein to certain extent, thus trafficking from the ER to the apoplast. Moreover, some studies [53–56] have also suggested that the attachment of GalNAc to substrate proteins might also occur in regions of the ER (Additional file 3: Fig. S1), therefore we could consider to subject Sec-G-CSF to the *O*-glycosylation machinery.

3. *O*-Glycosylation of Sec-G-CSF.

**Fig. 5:**
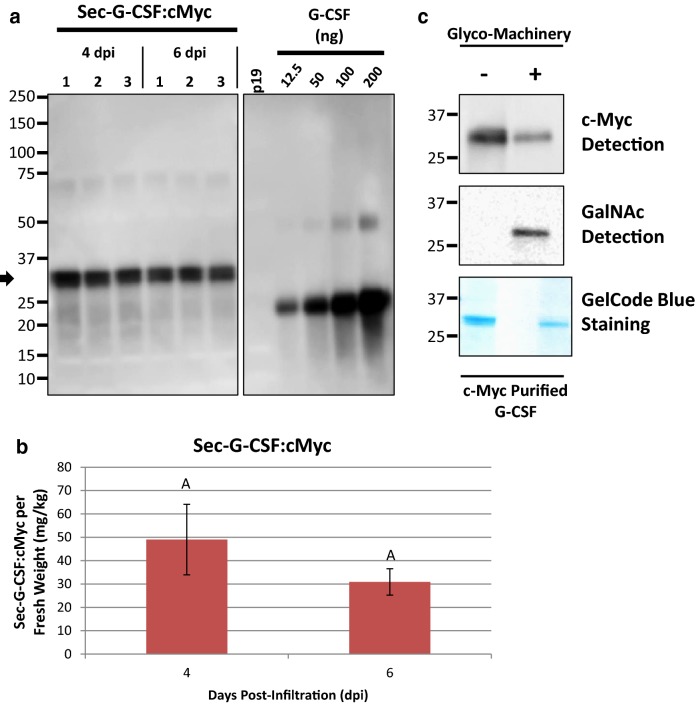
Western and Lectin Blot of *O*-glycosylated Sec-G-CSF. **a** Western blot detection of Sec-G-CSF:cMyc (Secretory variant, 28 kDa). Samples collected at 4 and 6 dpi. Four leaf discs from three different plants were collected for each sample. 20 µg TSP of PBS samples were loaded on the gel. Black arrow denotes monomeric Sec-G-CSF:cMyc. G-CSF: protein standard. Proteins were detected with G-CSF antibody. **b** Band quantification of Western blot detected Sec-G-CSF:cMyc. **c **c-Myc Detection: 0.25 µL eluent (Sec-G-CSF without glyco-machinery: -) after c-Myc purification; and 2.5 µL eluent (10x more than negative control) (Sec-G-CSF with glyco-machinery: +) after c-Myc purification. Detection using the c-Myc antibody. GalNAc Detection (Lectin Blot):: Equalized amounts of c-Myc purified Sec-G-CSF (without and with glyco-machinery: -, +). GalNAc detection using VVA-HRP. GelCode Blue: GelCode Blue Staining of c-Myc purified Sec-G-CSF (without and with glyco-machinery: -, +). Sec-G-CSF:cMyc co-expressed with p19 and glyco-machinery (∓) (O-Glycosylation machinery: UDP-GlcNAc/GalNAc epimerase, UDP-GlcNAc/GalNAc transporter, and GalNAc-glycosyltransferase) (OD_600_ ratios; Sec-G-CSF:cMyc: 0.5; p19: 0.1; GNT2: 0.2; epimerase/transporter: 0.1/strain). Columns denoted with a different letter are significantly different (*p* ≤ 0.05) using one-way ANOVA and followed by Tukey test. Error bars are standard deviation of the means

Once we confirmed that Sec-G-CSF was targeted for secretion, the Sec-G-CSF coding sequence was co-expressed with the *O*-glycosylation machinery (glyco-machinery) comprised by the *Y. enterocolitica* UDP-GlcNAc/GalNAc 4-epimerase, the *C. elegans* UDP-GlcNAc/GalNAc transporter, and the human GalNAc-Transferase 2, to allow the mammalian-specific GalNAc-*O*-glycosylation of G-CSF. In this case, the eYFP tag was replaced by a C-terminal c-Myc_4x_ tag (Sec-G-CSF:cMyc) for detection and purification purposes. Expression of Sec-G-CSF:cMyc was determined by western blot with a molecular weight of 28 kDa (Fig. 5a). Sec-G-CSF:cMyc reached accumulation levels of 49 mg/Kg F.W. and 31 mg/Kg F.W., at 4 and 6 dpi, respectively (Fig. 5b).

Once expression of Sec-G-CSF:cMyc was assessed, the secretory variant was then co-expressed with the *O*-glycosylation machinery. Sec-G-CSF:cMyc co-expressed with and without the *O*-glycosylation machinery was purified by c-Myc monoclonal antibody affinity chromatography, and analyzed via SDS-PAGE, c-Myc detection by western blot and GalNAc-*O*-glycosylation detection by lectin blotting (Fig. 5c). Sec-G-CSF:cMyc was detected both when co-expressed with or without the *O*-glycosylation machinery by SDS-PAGE and western blot using a c-Myc specific antibody (Fig. 5c). For detection of the *O*-glycosylated residue, the lectin *Vicia villosa* agglutinin conjugated with HRP (VVA-HRP), which is specific for GalNAc-*O*-T/S moieties, was used as probe (Fig. 5c). VVA-HRP reacted only with the Sec-G-CSF:cMyc co-expressed with the glycosylation machinery, but not with the Sec-G-CSF:cMyc expressed alone, indicating proper GalNAc-*O*-glycosylation of Sec-G-CSF:cMyc. To further corroborate the specific attachment of the GalNAc to the Thr-163 residue (Additional file 4: Fig. S4), Sec-G-CSF:cMyc co-expressed with and without the *O*-glycosylation machinery was purified and in-gel Trypsin/Chymotrypsin digested. A Trypsin/Chymotrypsin digestion system was used to generate a smaller peptide around the predicted *O*-glycosylation site that would be more amenable to MS/MS analysis. The resulting peptides were identified by nano LC–MS/MS and GalNAc attachment to the specific Thr-163 site was confirmed by the identification of an *N*-acetylhexosamine (HexNAc) derived from the peptide where the Thr-163 is localized, as indicated by the characteristic HexNAc product ion at *m/z* 204 (Additional file 4: Fig. S4; Additional file 5: Table S1). Nevertheless, Sec-G-CSF expressed alone could not be detected during the MS/MS analysis; moreover, analyses targeted to detect plant-specific post-translational modifications, such as proline hydroxylation and further plant-specific *O*-glycosylation, which are likely to occur during secretion of recombinant proteins, were not carried out in the present study. Thus, future studies will have to be carried out in order to fully characterize the recombinant protein.

## Discussion

Accumulation has been a major focal point for the production of recombinant proteins in plants [4, 38, 57, 58]. Although it may be assumed that post-translational modifications, such as glycosylation which might have an impact on protein stability or activity [6, 11], would be produced by plants and other eukaryotic expression platforms, most studies concerning expression of glycoproteins do not address this issue [9, 11, 16, 32, 59–63]. In this study, we address the feasibility of introducing the mammalian-specific mucin-type *O*-glycosylation to the pharmaceutical glycoprotein human G-CSF, in *N. benthamiana* plants via transient expression.

G-CSF is commercially produced in *E. coli* and CHO cells, well-established expression platforms. The bacterial system is capable of producing the recombinant protein in its mature form, with an extra N-terminal Met, and the protein is not glycosylated [35, 64]. The mammalian system is capable of decorating the recombinant protein with Gal-GalNAc-*O*-glycosylation with up to two sialic acids, at the single Thr residue reported to be mucin-type *O*-glycosylated in humans [35, 65].

Studies addressing the glycosylation differences between these two commercially available G-CSF’s have determined that lack of glycosylation has an impact on the protein’s susceptibility to degradation, decreasing the biological activity of the cytokine, and thus an increased amount of the non-glycosylated form is required to obtain a similar biological effect as that obtained with the glycosylated form [33, 34, 37]. Other studies have also suggested that the lack of glycosylation might promote protein aggregation, which would hamper the biological activity of the protein [30, 66–68].

Due to the pharmaceutical relevance of this glycoprotein, several alternative production platforms have been considered. Among these platforms, yeasts have been explored due to the secretory system they possess, which is similar to that of higher order eukaryotes [29, 69]. Recombinant hG-CSF has been successfully expressed in *Saccharomyces cerevisiae* and *Pichia pastoris* [29, 31, 66], but recent analysis reported that the recombinant *P. pastoris*-produced protein was not mucin-type *O*-glycosylated. Instead, the *O*-glycosylation Thr site used in mammalian cells was mannose *O*-glycosylated [70]. In the case of the *S. cerevisiae*-derived recombinant protein, glycosylation was apparently absent [66]. Furthermore, the yeast-derived recombinant protein was found mainly as multimers, which were not biologically active and had to be denatured and re-natured to recover proper protein folding and regain biological activity. The issue with multimerization is believed to result from lack of proper protein folding, as it has also been observed when G-CSF was expressed using the filamentous fungus *Aspergillus niger*, where glycosylation could not be determined [67].

Recently, G-CSF production has been achieved in tobacco and rice suspension cells [59, 62] and tobacco plants [60], targeting the protein to the secretory pathway; ER-retained in BY-2 cells [61]; in lettuce chloroplasts [32]; and transiently in *N. benthamiana* [63]. These studies do not report multimerization as it was observed in the case of the yeast and *A. niger* reports, and the protein was proved to be biologically active. However, glycosylation was not addressed in any of the previous studies.

In the present study, we have successfully expressed recombinant GalNAc-*O*-glycosylated G-CSF in *N. benthamiana* plants. Mammalian-specific *O*-glycosylation of the plant-derived G-CSF was achieved by targeting the recombinant protein to the secretory pathway, using its native secretory signal peptide and co-expressing it with a GalNAc-*O*-glycosylation machinery [6]. The recombinant secretory G-CSF (Sec-G-CSF) was confirmed to be GalNAc-*O*-glycosylated via lectin blot. Further mass spectrometry analysis corroborated the proper GalNAc-*O*-glycosylation at the single Thr residue reported to be mucin-type *O*-glycosylated in the native protein. Although plant-specific glycosylation is known to occur on proteins targeted to the secretory pathway, more specifically *O*-glycosylation at hydroxylated prolines, analyses in the present study were not addressed to characterize such post-translational modifications. Thus, further analytical studies will be required to fully characterize the glycosylation profile of the recombinant protein, as well as to determine glycosylation ratios. Nevertheless, corroboration of proper GalNAc-*O*-glycosylation of the Sec-G-CSF variant highlights the feasibility to further elongate the GalNAc-*O*-glycosylation moiety and imitate native mucin-type *O*-glycosylation. This objective would require the co-expression of additional glycosyltransferases and other proteins, as it has been demonstrated by recent studies where the GalNAc-*O*-glycosylated sites of recombinant proteins transiently expressed in *N. benthamiana* plants, were further elongated with Galactose and sialic acid [14–16, 71, 72].

In relation to multimerization, Sec-G-CSF did not seem to multimerize, in contrast to the other two variants targeted to the cytoplasm or expressed as ER-derived protein bodies (Cyt-G-CSF and Zera-G-CSF, respectively) (Figs. 2, 3; Additional file 3: Fig. S3). As reported previously by other studies where recombinant G-CSF was expressed in yeast and *A. niger* [29, 31, 66, 67], multimerization had a negative impact on biological activity and it has been suggested that it is caused by the lack of proper protein folding, by escaping the ER processing [67]. The fact that Sec-G-CSF was glycosylated does not only support the evidence that the native secretory signal peptide did indeed target the recombinant protein to the secretory pathway, since the GalNAc-Transferase is known to locate and exert its activity in the Golgi apparatus or in subregions of the ER (Additional file 3: Fig. S1) [15, 49–52], but localization of G-CSF in the secretory pathway also suggests proper protein processing by the ER, which together with further glycosylation, might decrease the nature of oligomerization of G-CSF, as suggested by other studies [30, 66–68]. Lack of multimerization and proper glycosylation are positively related to biological activity in this case, indicating the potential of the expression platform used in this project for the production of this glycoprotein. Nevertheless, proper studies related to the impact of glycosylation and ER processing to multimerization and biological activity will have to be carried out in future work.

It must also be noticed that the accumulation levels obtained with the secretory G-CSF were the lowest in comparison to the other two variants (Cyt-G-CSF and Zera-G-CSF). Co-expression with the glycosylation machinery decreased the accumulation levels of Sec-G-CSF:cMyc even more (approximately 10–30 × decrease was observed during the experiments, data not shown) (Fig. 5). Such decrease was expected since the expression of several recombinant proteins and their respective markers would cause an exhaustive usage of the translation machinery, therefore it would be advisable to generate a single multiple expression cassette containing only the required transgenes, assuring at the same time that all genes get expressed in the same cells [16]. Likewise, future subcellular relocalization of the recombinant G-CSF together with glycosyltransferases, as well as developing stable transgenic lines with the corresponding glycosylation machinery, should also be considered as an attempt to increase accumulation levels, while allowing the production of mucin-type *O*-glycosylated G-CSF in plants [4].

## Conclusions

In conclusion, we have successfully expressed GalNAc-*O*-glycosylated G-CSF in *N. benthamiana* plants via agroinfiltration, offering an alternative system for the production of this pharmaceutical protein with its native mammalian-specific post-translational modification. Further studies would be required to explore elongation of the glycosylation moiety and approaches to increasing accumulation of this recombinant glycoprotein.

## Additional files

**Additional file 1: Figure S3.** Impact of extraction buffer on G-CSF. A Western Blot detection of Sec-G-CSF:eYFP and Cyt-G-CSF:eYFP extracted with PBST^0.1 %^ (gels on the left) or under reducing and denaturing extraction conditions (SDS-DTT) (gels on the right). **B** Band quantification of Western blot detected proteins. Samples collected at 4, 6 and 8 dpi. Four leaf discs from different leaves were collected from each biological sample. 20 μL TSP of PBST^0.1 %^ treated sample or equivalent volume of SDS-DTT treated sample were loaded on the gel. Black arrows denote monomeric G-CSF:eYFP variant. p19: negative control. eGEHK: protein standard. Proteins were detected with GFP antibody. Band quantification of Western blot detected proteins (Graph). Columns denoted with a different letter are significantly different (*p* ≤ 0.05) using one-way ANOVA and followed by Tukey test. Error bars are standard deviation of the means

**Additional file 2: Figure S2.** Secretory and Cytoplasmic G-CSF can be extracted with a PBS-based extraction buffer, but Zera-G-CSF can only be extracted under denaturing and reducing conditions. Western Blot detection of G-CSF:eYFP variants transiently expressed in *N. benthamiana* plants. Samples collected at 4 dpi. Upper panel: Plant 1; Lower panel: Plant 2. Sec-G-CSF:eYFP (Secretory variant, 49 kDa), Cyt-G-CSF:eYFP (Cytoplasmic and mature protein variant, 49 kDa), Zera-G-CSF:eYFP (Zera fused variant, 61 kDa), eYFP (Cytoplasmic eYFP, 29 kDa), p19 negative control. Four leaf discs from two different plants were collected for each sample. 50 µg TSP of PBST samples or equivalent volume of SDS-DTT samples were loaded on the gel. Black arrows denote Zera-G-CSF:eYFP. Gray arrows denote Sec-GCSF-:eYFP and Cyt-G-CSF:eYFP. Red asterisks denotes a faint band corresponding to Zera-G-CSF:eYFP extracted with PBST extraction buffer and its corresponding proper extraction with SDS-DTT extraction buffer. Proteins were detected with anti GFP

**Additional file 3: Figure S1.** Compartmentalization of GalNAc attachment to target proteins in the secretory pathway. GalNAc-Transferases are traditionally referred to be localized in the Golgi Apparatus (Cis-Golgi), but recent studies suggest also ER localization, thus proposing attachment of GalNAc in subregions of ER and proximal Golgi compartment. GalNAc-T, GalNAc transferase. ER, Endoplasmic Reticulum. ERGIC, intermediate ER-Golgi Compartment. Grey box, GalNAc

**Additional file 4: Figure S4.** MS/MS identification of glycosylated Sec-G-CSF:cMyc-derived peptide. A Schematic illustration of released peptide after Trypsin/Chymotrypsin in gel digestion of c-Myc purified Sec-G-CSF:cMyc expressed alone or co-expressed with the *O*-glycosylation machinery. Native glycosylation site (Thr-163) is denoted by underlining. **B** Extracted ion chromatograms of predicted QQMEELGMAPALQPTQGAMPAFASAF peptide derived from Sec-G-CSF:cMyc expressed alone (upper panel), not being detected; or co-expressed with the *O*-glycosylation machinery (lower panel), where it was detected. **C** MS/MS of c-Myc purified and Trypsin/Chymotrypsin in gel digested Sec-G-CSF:cMyc-derived peptide (QQMEELGMAPALQPTQGAMPAFASAF) co-expressed with the *O*-glycosylation machinery, showing identified *b*- and *y*-ions. Modified Thr-163 is marked in red. Complete list of detected product ions is shown in Supplementary Table S1

**Additional file 5: Table S1.** *y*- and *b*- product ions detected in GalNAc-*O*-glycosylated QQMEELGMAPALQPTQGAMPAFASAF derived peptide and associated mass errors
