# Supplementary figures and images for: Transient co-expression with three O-glycosylation enzymes allows production of GalNAc-O-glycosylated Granulocyte-Colony Stimulating Factor in N. benthamiana
Source: Plant Methods. 2018 Nov 6;14:98. doi: 10.1186/s13007-018-0363-y (PMC6219069; doi:10.1186/s13007-018-0363-y)

Figure S3

A)

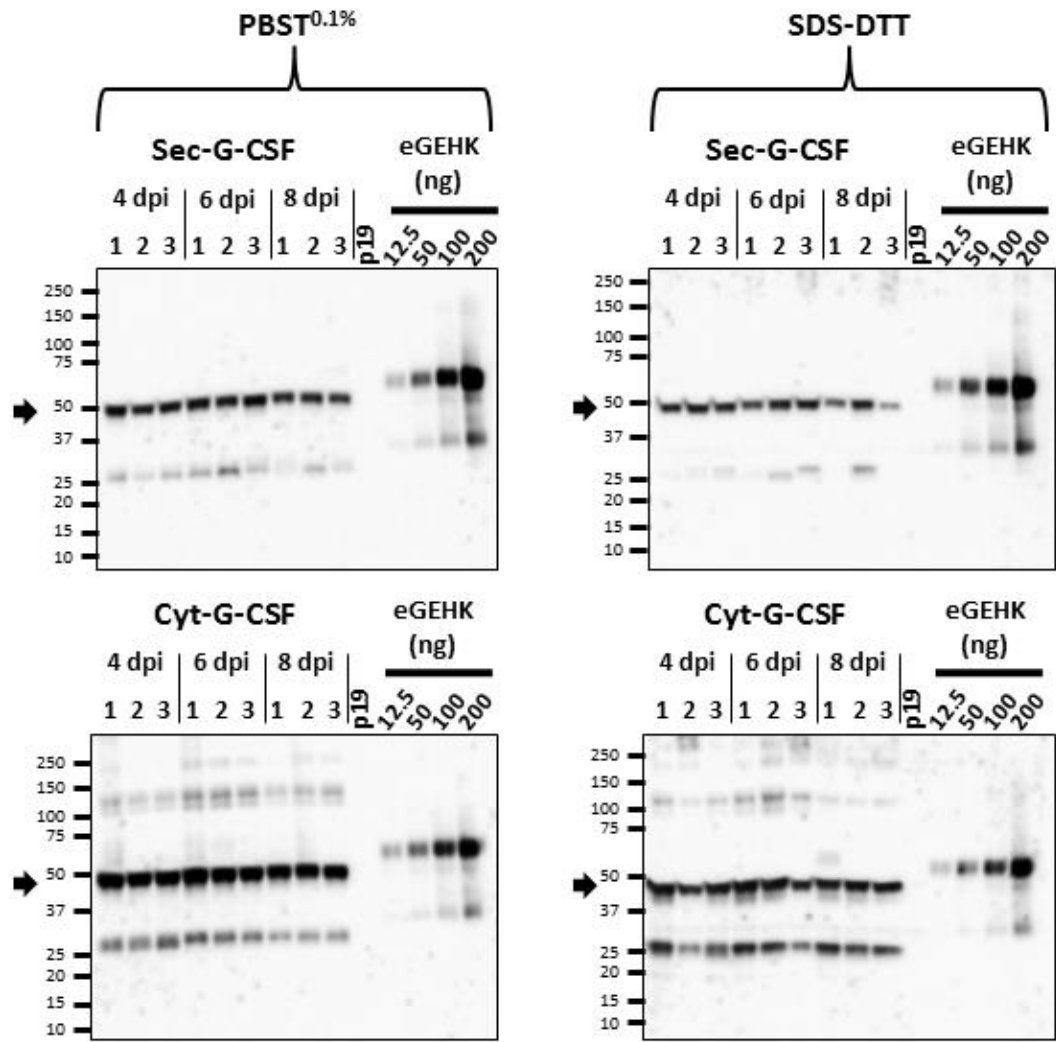

B)

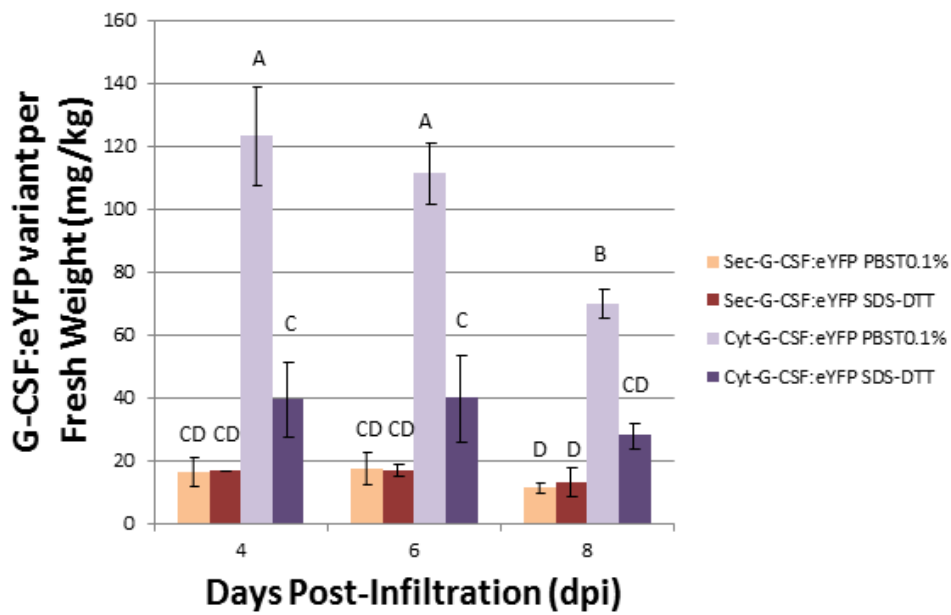

Supplement: Supplementary file 1 — Additional file 1: Figure S3. Impact of extraction buffer on G-CSF. A Western Blot detection of Sec-G-CSF:eYFP and Cyt-G-CSF:eYFP extracted with PBST0.1 % (gels on the left) or under reducing and denaturing extraction conditions (SDS-DTT) (gels on the right). B Band quantification of Western blot detected proteins. Samples collected at 4, 6 and 8 dpi. Four leaf discs from different leaves were collected from each biological sample. 20 μL TSP of PBST0.1 % treated sample or equivalent volume of SDS-DTT treated sample were loaded on the gel. Black arrows denote monomeric G-CSF:eYFP variant. p19: negative control. eGEHK: protein standard. Proteins were detected with GFP antibody. Band quantification of Western blot detected proteins (Graph). Columns denoted with a different letter are significantly different (p ≤ 0.05) using one-way ANOVA and followed by Tukey test. Error bars are standard deviation of the means [file 13007_2018_363_MOESM1_ESM.pdf]

Figure S2

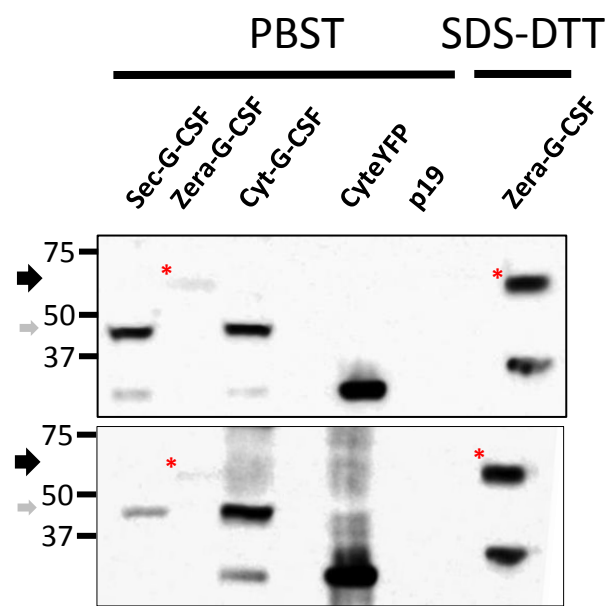

Supplement: Supplementary file 2 — Additional file 2: Figure S2. Secretory and Cytoplasmic G-CSF can be extracted with a PBS-based extraction buffer, but Zera-G-CSF can only be extracted under denaturing and reducing conditions. Western Blot detection of G-CSF:eYFP variants transiently expressed in N. benthamiana plants. Samples collected at 4 dpi. Upper panel: Plant 1; Lower panel: Plant 2. Sec-G-CSF:eYFP (Secretory variant, 49 kDa), Cyt-G-CSF:eYFP (Cytoplasmic and mature protein variant, 49 kDa), Zera-G-CSF:eYFP (Zera fused variant, 61 kDa), eYFP (Cytoplasmic eYFP, 29 kDa), p19 negative control. Four leaf discs from two different plants were collected for each sample. 50 µg TSP of PBST samples or equivalent volume of SDS-DTT samples were loaded on the gel. Black arrows denote Zera-G-CSF:eYFP. Gray arrows denote Sec-GCSF-:eYFP and Cyt-G-CSF:eYFP. Red asterisks denotes a faint band corresponding to Zera-G-CSF:eYFP extracted with PBST extraction buffer and its corresponding proper extraction with SDS-DTT extraction buffer. Proteins were detected with anti GFP [file 13007_2018_363_MOESM2_ESM.pdf]

Figure S1

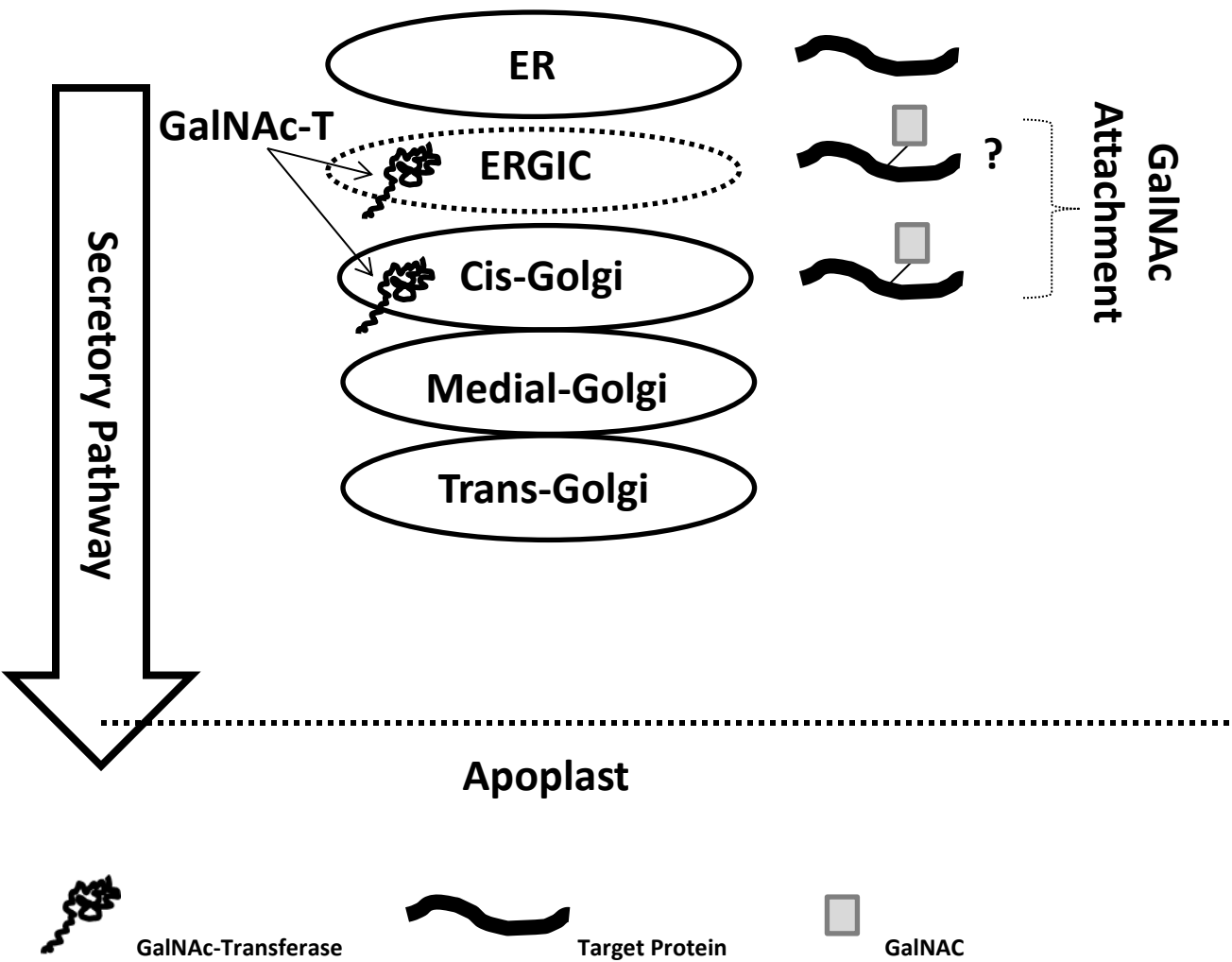

Supplement: Supplementary file 3 — Additional file 3: Figure S1. Compartmentalization of GalNAc attachment to target proteins in the secretory pathway. GalNAc-Transferases are traditionally referred to be localized in the Golgi Apparatus (Cis-Golgi), but recent studies suggest also ER localization, thus proposing attachment of GalNAc in subregions of ER and proximal Golgi compartment. GalNAc-T, GalNAc transferase. ER, Endoplasmic Reticulum. ERGIC, intermediate ER-Golgi Compartment. Grey box, GalNAc [file 13007_2018_363_MOESM3_ESM.pdf]

Figure S4

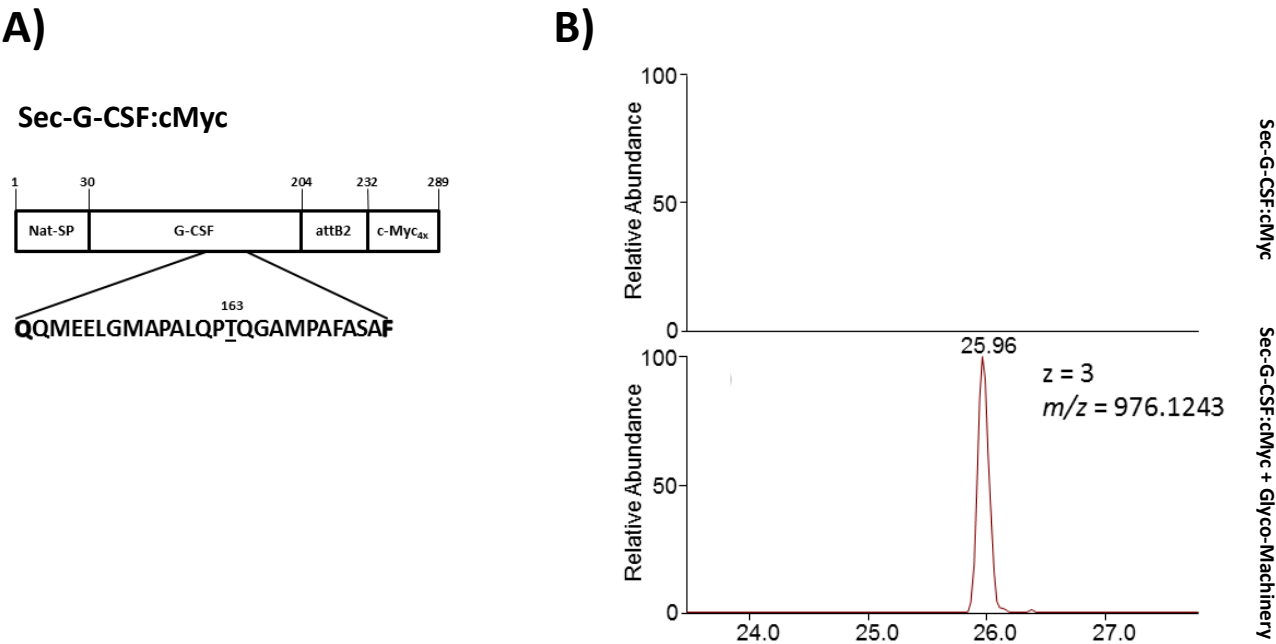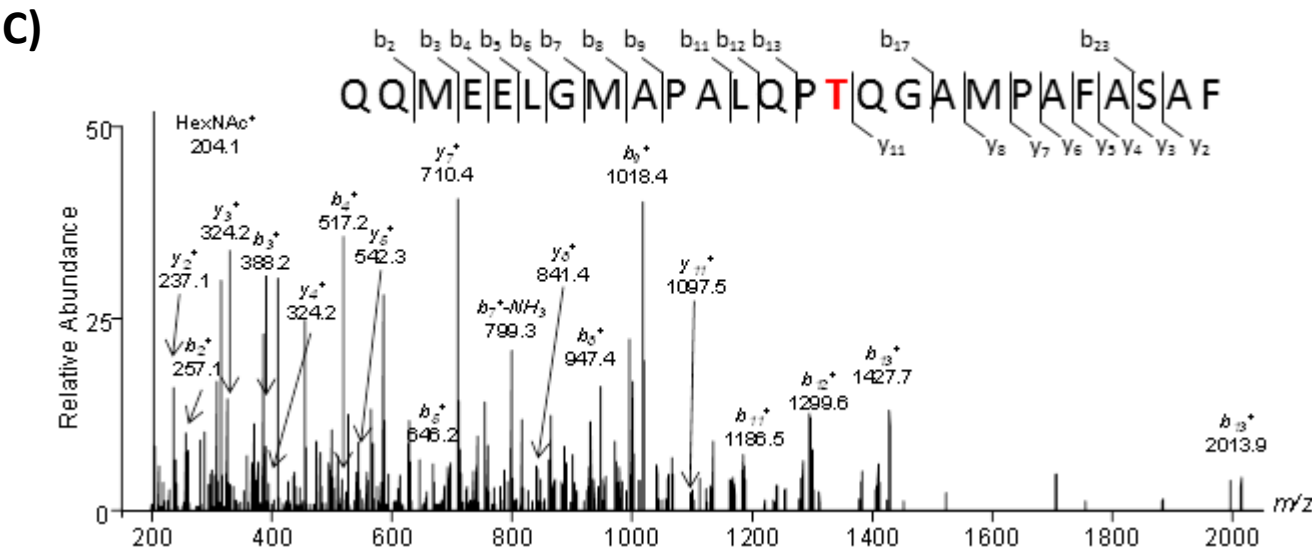

Supplement: Supplementary file 4 — Additional file 4: Figure S4. MS/MS identification of glycosylated Sec-G-CSF:cMyc-derived peptide. A Schematic illustration of released peptide after Trypsin/Chymotrypsin in gel digestion of c-Myc purified Sec-G-CSF:cMyc expressed alone or co-expressed with the O-glycosylation machinery. Native glycosylation site (Thr-163) is denoted by underlining. B Extracted ion chromatograms of predicted QQMEELGMAPALQPTQGAMPAFASAF peptide derived from Sec-G-CSF:cMyc expressed alone (upper panel), not being detected; or co-expressed with the O-glycosylation machinery (lower panel), where it was detected. C MS/MS of c-Myc purified and Trypsin/Chymotrypsin in gel digested Sec-G-CSF:cMyc-derived peptide (QQMEELGMAPALQPTQGAMPAFASAF) co-expressed with the O-glycosylation machinery, showing identified b- and y-ions. Modified Thr-163 is marked in red. Complete list of detected product ions is shown in Supplementary Table S1 [file 13007_2018_363_MOESM4_ESM.pdf]
