# Supplementary material for: Transient co-expression with three O-glycosylation enzymes allows production of GalNAc-O-glycosylated Granulocyte-Colony Stimulating Factor in N. benthamiana
Source: Plant Methods. 2018 Nov 6;14:98. doi: 10.1186/s13007-018-0363-y (PMC6219069; doi:10.1186/s13007-018-0363-y)
Supplement: Supplementary file 5 — Additional file 5: Table S1. y- and b- product ions detected in GalNAc-O-glycosylated QQMEELGMAPALQPTQGAMPAFASAF derived peptide and associated mass errors [file 13007_2018_363_MOESM5_ESM.pdf]

# Table S1

| ion | *m/z* | ppm | ion | *m/z* | ppm |
|---|---|---|---|---|---|
| *b2* | 257.12548 | -1.0883 | *y2* | 237.12408 | -0.6845 |
| *b2-NH3* | 240.09944 | -1.5415 | *y3* | 324.15616 | -0.7584 |
| *b3* | 388.16653 | -1.6344 | *y3-H2O* | 306.14578 | -0.9825 |
| *b3-NH3* | 371.13919 | -0.7907 | *y4* | 395.19284 | -0.3406 |
| *b4* | 517.21539 | -7.8931 | *y4-H2O* | 377.18256 | -0.6562 |
| *b4++* | 259.10741 | 0.08 | *y5* | 542.26090 | 2.9654 |
| *b4-H2O* | 499.19873 | -1.8305 | *y5-H2O* | 474.24353 | -8.8303 |
| *b4-NH3* | 500.181763 | -0.7627 | *y6-H2O* | 605.28046 | -5.2565 |
| *b5* | 646.25013 | -0.07 | *y7* | 710.35080 | -2.7156 |
| *b5-H2O* | 628.23822 | 1.2798 | *y7-H2O* | 676.31329 | -0.9935 |
| *b5-NH3* | 629.22632 | -2.7184 | *y8* | 841.39130 | 2.9943 |
| *b6* | 759.33844 | -4.2399 | *y8-H2O* | 733.33893 | -5.1282 |
| *b6-H2O* | 741.32812 | -4.525 | *y11* | 1097.50800 | -2.98221 |
| *b6-NH3* | 742.311462 | -3.8624 | *y13-NH3* | 1409.65723 | -1.43794 |
| *b7* | 816.354858 | 0.7416 | | | |
| *b7-NH3* | 799.33258 | -3.4806 | | | |
| *b8* | 947.39948 | -3.3751 | | | |
| *b8-H2O* | 929.38800 | -2.5005 | | | |
| *b8-NH3* | 930.37634 | -6.7428 | | | |
| *b9* | 1018.4365 | -3.262 | | | |
| *b9++* | 509.72036 | 0.27 | | | |
| *b9-H2O* | 1000.42694 | -4.241 | | | |
| *b10++* | 558.24667 | -0.115 | | | |
| *b11* | 1186.52039 | 2.714 | | | |
| *b12* | 1299.60278 | 4.417 | | | |
| *b13* | 1427.66675 | -1.048 | | | |
| *b13++* | 714.33659 | 0.08 | | | |
| *b14-H2O* | 1410.64880 | -6.80826 | | | |
| *b17* | 2013.92859 | -2.989 | | | |
| *b17-H2O* | 1995.91186 | 3.135 | | | |
